# Rumination’s Role in Second Victim Nurses’ Recovery From Psychological Trauma: A Cross-Sectional Study in China

**DOI:** 10.3389/fpsyg.2022.860902

**Published:** 2022-05-03

**Authors:** Lianrong Sun, Juan Deng, Jixin Xu, Xuchun Ye

**Affiliations:** ^1^Nursing College, Naval Medical University, Shanghai, China; ^2^Tianhua College, Shanghai Normal University, Shanghai, China; ^3^Academic Library, Shanghai Normal University, Shanghai, China

**Keywords:** nurse, second victim, psychological trauma, rumination, post-traumatic growth

## Abstract

**Background:** Nurses can experience psychological trauma after adverse nursing events, making it likely for them to become second victims (SVs). This negatively impacts patient safety and nurses’ development. This study aims to understand the status of psychological trauma and recovery of nurses as SVs in domestic China and examine the influencing mechanism of cognitive rumination during their recovery from psychological damage.

**Methods:** This was a cross-sectional survey. An online questionnaire was completed by 233 nurses from across China. Data were collected using Chinese versions of the Second Victim Experience and Support Evaluation Scale, the Incident-related Rumination Meditation Questionnaire, and the post-traumatic growth (PTG) Rating Scale. Descriptive statistics, correlation, and regression, as well as mediation analysis, were used for different analyses in this study.

**Results:** Participants experienced apparent psychological traumas (4.65 ± 0.5583) with a certain degree of PTG (76.18 ± 16.0040); they reported a strong need for psychological support (95.7%). Psychological trauma was positively and negatively correlated with rumination and PTG (*r* = 0.465, *p* < 0.001; *r* = −0.155, *p* < 0.05) respectively. Both psychologically impaired experience and rumination had significant predictive effects on participants’ PTG (both, *p* < 0.001). Nurses’ active rumination significantly mediated their psychological recovery from trauma to PTG (*p* < 0.05), but the effect of invasive rumination was not significant (*p* > 0.05).

**Limitation:** The specific manifestations of the mechanism of invasive rumination are not clarified in this study.

**Conclusion:** The present study investigated the psychological trauma of SV nurses as well as their support needs, and explored the role of cognitive rumination in the psychological repair and PTG of SV nurses. Results showed that SV nurses’ active rumination on adverse nursing events could promote their recovery from psychological trauma, but invasive rumination could not. This study provides a trauma-informed approach to care at the clinical level for nurses who experience psychological trauma caused by adverse events.

## Introduction

Nurses are the main forces serving public health and patient safety. In China, the number of registered nurses exceeds 4.7 million, accounting for more than 50% of the total number of health professionals on the mainland. However, according to the Chinese National Mental Health Development Report (2019–2020; Chinese Psychological Society; CPS, 2021), medical workers’ quality of mental health was quite alarming, particularly among nurses. The results showed that the mental health scores for nurses were significantly lower than the national average; 15.9–45.71% of nurses met the screening criteria for emotional problems; among them, those who reached the level of severe anxiety accounted for 9% (Fu et al., 2021).

Due to occupational risks associated with patient safety events, nurses are likely to experience psychological trauma, making them the second victim (SVs). SV refers to medical personnel who are physically and mentally injured during unexpected adverse medical events (Wu, 2000; Scott et al., 2010). They blame themselves for the patient’s adverse consequences, doubt their clinical ability, and encounter a series of mental health problems and career dilemmas (Wu and Steckelberg, 2012; Seys et al., 2013; Burlison et al., 2017). Some of them even experience long-term negative effects that are similar to post-traumatic stress disorder, with headaches, nightmares, uncontrollable event rumination, and even suicidal tendencies (Chan et al., 2018).

The SV phenomenon is widespread in medical institutions. Surveys showed that 84% of medical staff have experienced at least one patient’s unexpected death or serious injury in their career, 19% reported that they could not completely eliminate the negative effects of adverse events, and 67% acknowledged that their ability to diagnose and care had been seriously affected for 4 h after the incident (Merandi et al., 2017). Of the respondents, 43.3% reported that adverse events seriously affected their personal lives (Wolf et al., 2000). Compared to physicians, this issue was more apparent among nurses. Studies from Brazil, Singapore, and the Netherlands have shown that more than 50% of nurses who experienced adverse events reported psychological trauma and encounters with severe insomnia, guilt, self-blame, invasive rumination, prejudice and discrimination from colleagues, job burnout, and even career crises (Lewis et al., 2013; Chan et al., 2018; Bohomol, 2019; Burlison et al., 2021). Qualitative studies performed by Chinese researchers also yielded consistent results, confirming that nurses who experienced adverse events struggle with negative emotions such as worry and self-blame for long periods of time (Chen et al., 2019a; Han et al., 2020).

Many relevant studies have shown that adverse nursing events, especially those with severe consequences, were major traumatic events for the nurses involved, and the associated mental symptoms were similar to the acute psychological mechanism of post-traumatic disorder. According to the theory of social cognitive processing, social support can provide a supportive environment for the traumatic individual, thereby promoting their psychological wellbeing (Lepore and Greenberg, 2002). With the deepening of research, scholars have proposed a comprehensive model of post-traumatic growth (PTG; Tedeschi and Calhoun, 1996; Berger and Weiss, 2010), expounding the key role of good social support and role models, as well as the individual’s own positive response and cognitive schema changes on their PTG. A series of studies on this topic also found that the optimization of environmental support was based on an individual’s positive interpretation of traumatic events (Dekel and Nuttman-Shwartz, 2009). Studies on individuals with breast cancer, depression, and children and adults with earthquake experience have shown that cognitive rumination on their traumatic experience is critical for the cognitive processing of psychological recovery after trauma (Soo and Sherman, 2015; Zetsche et al., 2018). Cognitive rumination, also known as ruminant thinking, is defined as thinking about the causes and consequences of negative events as well as one’s emotional state during these events, repeatedly (Wu et al., 2018). One type of cognitive rumination is invasive rumination which refers to the unexpected invasion of traumatic events into an individual’s cognitive world. Active rumination, on the other hand, is when individuals consciously think about traumatic events repeatedly (Wu et al., 2018). Previous research has shown that cognitive rumination plays an important role in the later psychological growth of individuals after traumatic events, however, the cognitive processing of invasive rumination and active rumination differ (Stockton et al., 2011; García et al., 2015). Invasive rumination tends to involve focusing on the cognition of the negative aspects of traumatic events and increase individuals’ negative evaluations of traumatic events (Ehlers and Clark, 2000), leading to increases in anxiety, helplessness, and the aggravation of traumatic mood, thus reducing the possibility for psychological growth (Watkins and Moulds, 2005). In contrast, active rumination involves proactive thinking about trauma-related clues. It plays a positive role in alleviating post-traumatic psychological stress, helping individuals modify their negative thinking patterns regarding trauma, and reducing fear reactions. These changes alleviate trauma symptoms and increase the possibility of psychological growth (Cann et al., 2010; Wu et al., 2018).

Researchers have previously pointed out that the roles of invasive and active rumination are not completely distinct. The PTG model proposed by Tedeschi and Calhoun (2004) showed the mechanism by which rumination promotes individual PTG. This model proposes that it is not the traumatic event itself, but that the challenging of as individual’s core belief system by the experience of a traumatic event, which leads to the individual’s cognitive activities. Further, because traumatic events are shocking to individuals, invasive rumination would be the first type of rumination to occur after an individual’s belief system was challenged. This invasive rumination would provide a prelude to the individual’s active rumination, facilitating it and helping the individual reflect and construct meaning in their post-traumatic world (Cho and Park, 2013).

Invasive and active rumination have been shown to impact post-traumatic recovery and growth both positively and negatively, and thus should be investigated individually. Individual internal and environmental factors should also be taken in to consideration (Shallcross et al., 2016; Zhou et al., 2019b). For example, individual resilience, emotional regulation, and external social support are protective factors for an SV’s healing from psychological trauma. In May 2020, Fino et al. measured the PTSD, psychological resilience, emotion regulation strategies, social support perception, and PTG of 202 front-line medical staff in a COVID-19 disaster area in northern Italy. Subsequent data analysis found that after experiencing psychological pain, PTG was more significant in individuals with higher levels of psychological elasticity, more active emotion regulation strategies, and better perceived social support, but had no effect on individuals with low levels of psychological elasticity, emotion regulation, and social support (Fino et al., 2021a). However, it is not known whether cognitive rumination will also have such effect in the psychological repair of SV nurses.

Although most studies of SVs focused on their psychological impairment, a few also found that some SV nurses reported psychological recovery and growth after experiencing adverse nursing events. Some of them reported that they had become more rigorous and focused on the improvement of professional skills and developed a more positive attitude for coping with difficulties in their daily life (Vanhaecht et al., 2019; Han et al., 2020). Hence, it is important to explore the path of SV nurses’ recovery and growth from psychological trauma, based on the theory and methods of cognitive psychology. However, the mechanisms underlying such psychological repairment and growth are not yet clear. Therefore, the present study used a cross-sectional design, combining the perspective of cognitive psychology to advocate for individuals’ internal growth and verify the effect of cognitive rumination on SV nurses’ psychological impairment recovery and PTG. We focused on three main issues: What is the psychological damage and growth status of SV nurses? What are SV nurses’ needs for psychological support? What impact does rumination on the adverse events they experienced have in their recovery and growth after psychological trauma? The corresponding research hypotheses are:

*H1*: SV nurses’ psychological impairment levels as well as their demands for psychological support would be higher than the average for other nurses.

**Figure 1:**
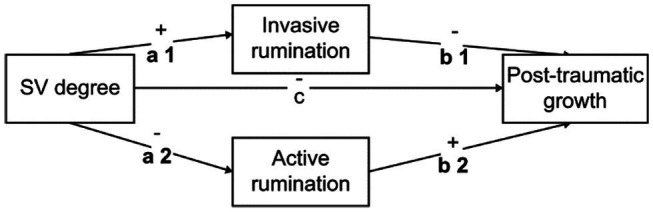
Hypothetical model: active and invasive rumination as bi-meditating variables. *a1* refers to the path coefficient of SV degree to invasive rumination, *b1* refers to the path coefficient of invasive rumination to PTG when SV degree and passive rumination are put into the model at the same time. Similarly, *a2* refers to the path coefficient of SV degree to active rumination, *b2* refers to the path coefficient of active rumination to PTG when SV degree and active rumination are put into the model at the same time. *c* refers to the total effect of SV degree on PTG.

*H2*: Cognitive rumination plays a regulatory role in the path from psychological trauma to PTG in SV nurses; active rumination promotes PTG while invasive rumination inhibits PTG (see the hypothetical model constructed in Figure 1).

## Materials and Methods

### Participants and Sampling

#### Sampling

**Figure 2:**
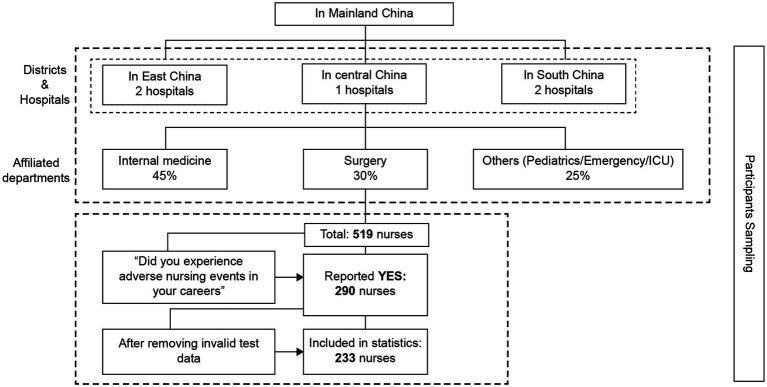
Participants sampling procedure.

We used the stratified sampling method. First, 519 Chinese nurses were recruited proportionally from 3 departments (Internal medicine, Surgery and others which include the Pediatrics, Emergency, ICU, etc.) of five general hospitals in Eastern, Central, and Southern China (see Figure 2). Subsequently, 290 (about 55.8%) nurses who reported experiencing adverse nursing events in their careers (personal experience and/or being a witness) were invited to participate in a survey investigating their SV psychological trauma and growth status, psychological support needs, and cognitive rumination. After rejecting the questionnaires that were incomplete or had invalid answers, the data of 233 nurses were included in the statistical analyses. The sampling efficiency was 80.3%.

#### Participant Inclusion and Exclusion Criteria

Nurses who have obtained the qualification certification and have been working continuously for more than 1 year in clinical practice were invited to participate in the present study. The exclusion criteria were as follows: Nurses who were temporarily on leave due to internships or advanced studies; nurses who previously suffered from or were currently suffering from mental disorders; and nurses who with unclear speeches.

### Survey Methods and Data Collection

The survey was conducted for 2 weeks in July 2020, and participants could only complete it once. After obtaining approval from the Institutional Review Board of the appropriate institution (IRB number: 2020-GZR-HS-003), we called the nursing department directors to explain the study’s purpose and seek their help with inviting the nurses in their respective hospitals to participate. After agreeing, the directors sent the electronic C-SVEST, C-ERRI, and C-PTGI questionnaires’ online link, which was set on the questionnaire star platform of China, to the nursing group. Nurses who consented anonymously filled out and submitted the questionnaires. The participants’ privacy and confidentiality were guaranteed strictly throughout the study.

### Measurements

#### Participants’ Basic Information and the Adverse Nursing Event

This included respondent demographics (gender, age, professional title, years of work experience, department, education, and marital status) and the characteristics of the adverse event they experienced (personally experienced or witnessed, time, frequency, type, and degree of harm to the patient).

#### The Second Victim Experience and Support Assessment Scale

The Chinese version of the Second Victim Experience and Support Assessment Scale (C-SVEST), which was translated and reconstructed by Chen et al. (2019b) from the SVEST (Burlison et al., 2017) was used to investigate psychological trauma and participants’ needs for support. Based on literature review and clinical experience, Burlison et al. (2017) developed SVEST, which has good reliability and validity. It has been widely used by scientific research teams in many countries worldwide to investigate and evaluate the psychological feelings and support needs of second victims. Based on the culture and nurses’ clinical practice in China, Chen et al. (2019b) translated SVEST into Chinese for the first time and conducted survey in domestic China. It contains six dimensions: psychological distress, physical distress, occupational difficulties, colleague support, management support, and family or friend support. The first three dimensions investigated the psychological, physical, and professional difficulties of SV nurses due to patient safety events, and the last three dimensions investigated SV nurses’ perceptions of support from colleagues, managers, family, and friends. There are 24 items in total, with scores ranging from 1 (strongly disagree) to 5 (strongly agree). The higher the score, the more negative the SV’s psychological experience is. The Cronbach α coefficient of the scale calculated based on the data of the present study research is 0.809, and the Cronbach α coefficients of subscales for psychological distress, physical distress, occupational difficulties, colleague support, management support, and family/friend support are 0.919, 0.942, 0.894, 0.798, 0.893, and 0.886, respectively.

#### The Event-Related Rumination Inventory

The Chinese version of the Event-Related Rumination Inventory (C-ERRI), translated and reconstructed by Dong et al. (2013) from the ERRI (Cann et al., 2011), was used to survey participants’ cognitive rumination after experiencing their adverse nursing events. According to the PTG model proposed by Tedeschi and Calhoun (2004), Cann et al. (2011) constructed a two-dimensional questionnaire for ruminant meditation (ERRI). The questionnaire has been used for the cognitive assessment of people experiencing traumatic events such as high-stress life event experiencers, Hurricane Katrina survivors, and HIV-infected people. It was confirmed to have good reliability and validity. Dong et al. (2013) were the first to create a Chinese version of the ERRI and confirmed that it can effectively measure the post-traumatic cognitive status of Chinese subjects. It has 20 items and two dimensions (active and invasive rumination), each consisting of 10 questions. The former tests the degree of active recall and thinking of traumatic events, and the latter tests the degree of forced flashbacks of traumatic events. It is scored using a 4-point Likert scale ranging from 0 (never had such an idea) to 3 (often had such an idea). The higher the score, the more intense the SV nurses’ rumination. The Cronbach α coefficient of the scale in the present study is 0.975, and the Cronbach α coefficients of the for invasive and active rumination subscales are 0.968 and 0.952, respectively.

#### Post-traumatic Growth Inventory

The Chinese version of the post-traumatic growth inventory (C-PTGI) was used to investigate nurses’ growth from the experience of adverse nursing events. The scale was translated and revised by Wang et al. (2011) from the Posttraumatic Growth Inventory (PTGI; Tedeschi and Calhoun, 1996). Tedeschi and Calhoun (1996) developed the PTGI, which was translated and revised by Germany, Japan, Netherlands, Spain, and Bosnia and Herzegovina. It was confirmed to have good reliability and validity. Wang et al. (2011), authorized by Professor Tedeschi, revised PTGI into a simplified Chinese version (C-PTGI) and researched Chinese subjects. It consisted of 21 items and five dimensions, including life perception, personal strength, new possibilities, relationships with others, and self-alteration. The scale used a 6-point Likert scale, with each item scored from 0 (no changes experienced) to 5 (a lot of changes experienced). Total scores range from 0 to 105 points, and a higher total score indicates greater growth after experiencing adverse nursing events. The Cronbach *α* coefficient of the scale in the present study is 0.956, and the Cronbach *α* coefficients of the subscales for life perception, personal strength, new possibilities, relationships with others, and self-alteration are 0.862, 0.774, 0.867, 0.777, and 0.776, respectively. Considering the purpose and content of the present study, we slightly adjusted the expression of some sentences while maintaining the original structure and the dimensions of the C-PTGI.

### Statistical Methods

SPSS 16.0 and Mplus 7.0 were used for statistical analysis. Descriptive statistical analysis was performed based on the demographic information and C-SVEST survey data. Then, correlation and regression analyses were performed in SPSS 16.0, and the mediation model was employed to verify the C-SVEST, C-ERRI, and C-PTGI data in Mplus 7.0. Such analyses were used to understand the status of psychological trauma and growth of SV nurses and to examine the influence path of active and invasive rumination on SV nurses’ psychological growth after experiencing adverse nursing events.

## Results

### Participants’ Basic Information

A total of 233 effective data items were sorted and analyzed. Among the subjects, 96.6% were female, aged 31–60 (41.53 ± 6.37 years); the details of other demographic information are shown in Supplementary Table 1.

### Differences in SV Scores on Various Demographic Variables

According to the SV scale scoring method, the two types of continuous data are assigned with 3 points as the dividing point, performed categorical conversion of the continuous data to label 3 points or more was HIGH Score SV, and 2.99 points or less was LOW Score SV. Then, using Crosstabs to analysis the subject’s SV score difference in their demographics. The result indicated that no significant difference related to gender, age, hospital level, department, working years, professional title, marital status, or employment type. The results of the mean difference test showed that there were no significant differences between groups in the PTG score of each demographic variable. Take this as a logical basis for subsequent inference statistics.

### Status of SV Nurses’ Psychological Trauma, Rumination, and PTG

As shown in Supplementary Table 2, the SV nurses had the highest scores in the psychological distress dimension, followed by physical distress; the scores for “colleague support” and “management support” were the lowest. Furthermore, the scores of the five items of the psychological distress dimension were listed in the descending order as follows: worried (4.65 ± 0.78112), upset (4.60 ± 0.688), miserable (4.55 ± 0.770), embarrassed (4.54 ± 0.748), and deep self-blame (4.53 ± 0.788).

Moreover, the total cognitive rumination score of the SV nurses was slightly greater than the median, and there was no significant difference between active and invasive rumination. In addition, the total PTG score and the scores for each dimension were higher than the median value.

### SV Nurses’ Support Needs

In the present survey, only 26.6% (62) of participants reported that they had heard of the concept of “second victim.” The SV nurses’ needs for different support approaches and their cumulative frequency were calculated according to the SVEST scoring rules (Burlison et al., 2017). As shown in Supplementary Table 3, the need for psychological support was the second highest (95.7%).

### Relations Between SV Nurses’ Psychological Trauma, Rumination, and PTG

#### Correlation and Regression Analyses

As shown in Supplementary Table 4, there was a significant correlation among the three variables. Based on such result, combined with the findings about the relationships among individuals’ PTSD, rumination, and PTG in the existing literature, we assumed that SV psychological trauma and rumination would have a significant predictive effect on their PTG. Therefore, multiple regression analysis was performed, taking the SV’s psychological trauma and cognitive rumination as predictors and PTG as the outcome variable. The results are presented in Supplementary Table 5. The SV’s psychological trauma had a significant negative predictive effect, while the active rumination had a significant positive predictive effect, on PTG.

#### Mediation Models Analysis

**Figure 3:**
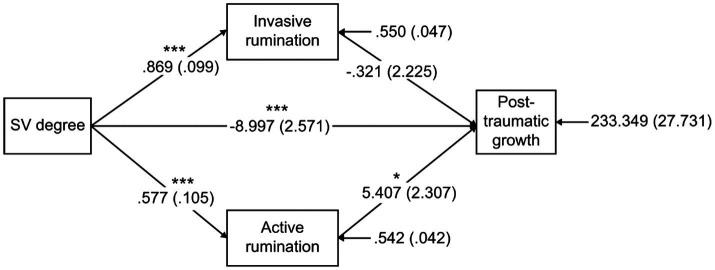
Tests of the dual mediating effect model of active and invasive rumination. For the data on the arrow between variables in the figure, the path estimate coefficients are outside the brackets, and the corresponding standard errors are inside the brackets. For the data with arrows pointing to variables such as active rumination, active rumination and PTG, the corresponding residuals are outside the brackets, and the standard errors are inside the brackets.

In order to test hypothesized mediational model in Figure 1, where active and invasive rumination mediated the effect of SV psychological trauma on their PTG. We calculated bias-corrected 95% CIs and bootstrap values for the indirect effect using 1,000 sample bootstrapping with the process procedure (Hayes, 2013; Wen and Ye, 2014). The results in Figure 3 show that the mediating effect of invasive rumination was not significant, while the mediating effect of active rumination was significant. The effect size|a2b2/c’| = 0.3469.

## Discussion

### SV Nurses Had Obvious Psychological Trauma and Were in Urgent Need of Psychological Support

The results of the present study indicate that participants suffered considerable amounts of psychological and physical distress and needed psychological support. There is plentiful research showing that nurses experienced physical and psychological disorders such as insomnia, sharp loss of appetite, anxiety, worry, or panic after experiencing adverse nursing events (Lewis et al., 2013; Chen et al., 2019a). Some of them also encountered difficulties in their career development (Seys et al., 2013). The present findings provide new evidence in this field, as more than 55% of participants who reported they had experienced or witnessed adverse nursing events during their careers suffered obvious psychological and physical distress. Among all the factors, negative feelings such as fear, depression, regret, stress, and self-blame were all scored above 4.5 (5 is the highest value), and physical distress such as insomnia and poor appetite were also rated above 4. In terms of support needs, although the participants had better feelings about receiving support from colleagues, managers, relatives, and friends after experiencing adverse nursing events (the scores were all below median points), the survey indicated they expected other psychological support as well. Of the SV nurses, 95.7% expected to receive confidential emotional support, which was second only to “expect to receive guidance and advice for future work after experiencing adverse events” (96.1%). Moreover, the SV nurses’ reporting requirements for obtaining legal advice on adverse events, participating in understanding the progress of events, and advancing the “exemption” cultures in the hospital were all higher than 90%. This finding is consistent with the results of a recent systematic review in domestic China (Chen et al., 2019a; Lin et al., 2020).

The SV nurses did not feel “practical distress” in the present study, and the scores of related items were not significantly different from the median. This finding was inconsistent with SV research results from Europe and America, which showed that SVs encountered rigorous career development crises (Seys et al., 2013). However, the consistent findings of domestic Chinese research have indicated that although the experience of adverse events will have a certain impact on their career development, it is not common for medical staff, as SVs, to suffer from career dilemmas (Chen et al., 2019a). As for such differences in domestic and foreign research, we considered the “practical distress” to SVs might be related to differences in Eastern and Western cultural backgrounds and medical systems. We know from existing domestic studies that SV nurses could receive active support for handling the incident and responding appropriately; they dealt with patients’ injuries promptly with the help of the matrons and the head of the nursing department and improved the follow-up nursing process (Chen et al., 2019b, 2020). However, the SV nurses had low approbation for frequently asking about “(event) Reporting on presentation,” and “Related leaders repeatedly asked about the incident and asked me whether I was responsible, but no one asked me whether I could bear all this” (Han et al., 2020; Wei et al., 2020). Such findings indicated that receiving psychological support from management was crucial to SV nurses, especially in the forms of emotional empathy and non-punishment culture.

### SV Nurses’ Recovery Depends on the Degree of Psychological Trauma and Rumination on the Adverse Event

The present study found that the PTG self-scores of SV nurses were higher than the median, indicating that they experienced psychological growth after the traumatic adverse event. This is consistent with previous research results from survivors of other types of traumas (Wu et al., 2018). Positive psychology argues that the more constructive coping path for traumatized individuals was to activate their internal recovery mechanism in addition to support from the external environment to obtain PTG. Whether such growth occurs is related to the degree of the individual’s trauma and rumination of the event (Tedeschi and Calhoun, 2004; Roley et al., 2015). In the present survey, there was a significant positive correlation between PTG and SVs’ rumination, but a significant negative correlation with the SVs’ psychological trauma. Subsequently, the multiple regression analysis results showed that SVs’ psychological experience had a significant negative predictive effect on PTG, while rumination had a significant positive predictive effect. This indicated that the stronger the experience of psychological trauma the SV nurses had, the less likely they were to have PTG later; however, ruminating on the event was beneficial for their PTG. This was consistent with Zhou et al.’s (2019a) research on earthquake survivors, where they found that rethinking the cause and process of the trauma event and the emotional awareness it caused could alleviate the survivor’s negative feelings and promote their PTG.

### Active and Invasive Rumination Play Different Roles in PTG

Studies in cognitive psychology have shown that active and passive invasiveness play different roles in an individual’s psychological recovery process. Active rumination promotes PTG, while invasive rumination hinders it (Tedeschi and Calhoun, 2004). The present survey showed that the SV nurses generally ruminated on the adverse nursing events they had experienced, and the degree of active and invasive rumination was roughly equivalent. Therefore, the core question was: *Do both types of rumination play the same role in promoting PTG?*

To address this question, the present study constructed a dual mediator variable model with active and invasive rumination as mediators (Figure 1). Data analysis results showed that the mediating effect of invasive rumination was not significant. In contrast, active rumination partially mediated the relationship between SVs’ psychological trauma and their PTG. This result indicates that the two forms of cognitive rumination played different roles in the process from psychological trauma to self-growth. That is, psychologically traumatic experiences could positively promote SVs’ PTG through active rumination, while invasive rumination did not have such a promotional effect.

Similar conclusions have been obtained from studies based on other types of trauma survivors; that is to say, active rumination overcomes fear responses and promotes constructive responses through individuals’ active understanding of traumatic events and then supports individuals’ growth after trauma. In contrast, invasive rumination tends to negatively interpret the traumatic event and focuses on its negative aspects, which leads to experiencing more anxiety, tension and, ultimately, leads to the aggravation of the psychological trauma and reduces the possibility of growth (Tedeschi and Calhoun, 2004). In the present study, active rumination’s promoting role in SV nurses’ process from psychological trauma to self-growth was confirmed, but no negative inhibitory effect on PTG was found for invasive rumination. A few studies have found that, although adverse events cause individuals to suffer psychological impairment and face professional difficulties, they also present opportunities to promote individuals’ growth. Although nearly 90% of people reported that they had a clear memory of the incident even after many years and failed to fully overcome the trouble it caused, 10% of people still recognized that they learned lessons from the incident and became stronger (Scott et al., 2009; Van Gerven et al., 2016). Similar evidence has been obtained from Chinese studies. Most of the SV nurses who experienced adverse nursing events that had not caused serious patient harm regarded such experiences as “an alert” and an “important learning opportunity in life”; they admitted that they became more serious and careful later on. Some also pointed out that such experiences made them realize the importance of self-regulation and encouraged them to learn about it (Chen et al., 2019b; Wei et al., 2020).

In addition, there may be some indirect protective factors in SVS nurses’ working environments (Fino et al., 2020, 2021b). For example, in one of their studies during the COVID-19 pandemic, Fino et al. (2021b) found that enhancing the emotional links between isolated patients and their relatives can effectively improve the psychological pain of dedicated nurses. In a cross-sectional study in June 2020, they arranged for front-line COVID-19 medical staff (*n* = 107) to help isolated patients use remote video communication to speak with their families. It was found that compared with the nurses who did not do so, the nurses who assisted patients in home video communication reported the degree of their psychological distress (e.g., burnout, post-traumatic stress, anxiety, depression, and sleep difficulties) significantly reduced. Combining the present findings and the conclusions of previous research on SV nurses’ psychological rehabilitation, we believe that the nurses’ invasive rumination did not deepen the experience of psychological trauma, but instead slowed down the recovery process and prolonged the duration of the SVs’ immersion in their psychological trauma.

In terms of the finding that the effect of invasive rumination was not significant in the mediation model, we inferred that this may be due to the characteristics of traumatic events experienced by the study participants. As discussed in the Introduction, the mechanism of invasive rumination hindering the psychological repair and PTG of individuals was believed to be that it caused individuals to enlarge their negative cognition of traumatic events, causing an increase in negative emotions, aggravating the symptoms of PTSD and reducing the possibility of PTG. The research that supported this mechanism was mostly based on major events related to the personal safety of trauma victims (e.g., earthquakes, cancer, domestic violence, and sexual assault). In present study, for SV nurses, patient safety events were common in their work environments, and were not as sudden as the events described in previous research, and, in most cases, the consequences of these incidents did not threaten the lives of SVs. Thus, the impact and degree of these events were different from the major traumatic events in previous literature. Therefore, invasive rumination in the present study did not significantly hinder the PTG of SVs, but only prolonged the stage of psychological repair. It is also likely to be the transition stage before the active rumination of SVs (Cho and Park, 2013).

Existing literature has shown that some intervention methods combining group counseling with cognitive therapy, such as cognitive behavioral therapy or cognitive reappraisal, as well as the training of nurses to guide communication between patients and their families, could effectively guide traumatized individuals to actively ruminate and process their negative experiences, fostering PTG (Buhle et al., 2014; Zhou et al., 2019a; Nagulendran and Jobson, 2020; Fino et al., 2021b). Such interventions might also provide meaningful insights into SV nurses’ psychological recovery and growth processes.

### Implications for Nurse Managers

Based on a sample of nurses who had experienced adverse nursing events, this study investigated the status quo of their SV experience and examined the relationships between SVs’ degree of psychological impairment and cognitive rumination and their effects on PTG. The research focused on detecting participants’ growth path after psychological trauma, from the perspective of positive psychology. At the same time, these results facilitated a deep understanding of the specific content of SV nurses’ support needs. These findings could provide constructive and effective cues for supporting SV nurses’ practice. It is necessary for hospitals and nurse managers to systematically care for SV nurses’ mental health. They should consider developing interventions that help SV nurses form active ruminations to promote recovery and PTG.

### Limitations

This research has a limitation. Although the different mechanisms of the two forms of cognitive ruminations have been discovered, the specific manifestations of the mechanism of invasive rumination remain to be clarified. Future research should combine longitudinal research on SV nurses and experimental studies to deeply explore the path of the above-mentioned “demarcation trigger effect” and its mechanism.

## Conclusion

Based on the psychological impairment of SVs and their urgent need for psychological support, we reviewed existing literature on the performance and influencing factors of SVs as well as the impact of cognitive rumination on PTSD and PTG. We then constructed a hypothesis to test the role of cognitive rumination in SV psychological repair and PTG. The present study indicated that nurses who experienced adverse events would likely become SVs. Additionally, rumination regarding the events that led these nurses to become SVs played a key role in the repair their psychological distress and their PTG. Based on the correlation and regression analyses, SV nurses’ psychological trauma and rumination had a significant predictive effect on their PTG. The mediation effect analysis showed that active rumination completely mediated the relationship between SV psychological trauma and PTG, while invasive rumination had a partially mediating effect. This indicates that the active cognitive rumination on adverse nursing events of SV nurses could promote their recovery from trauma, while invasive rumination did not have such an effect.

## Data Availability Statement

The raw data supporting the conclusions of this article will be made available by the authors, without undue reservation.

## Ethics Statement

The studies involving human participants were reviewed and approved by Institutional Review Board of the Naval Medical University (IRB number: 2020-GZR-HS-003). The patients/participants provided their written informed consent to participate in this study.

## Author Contributions

LS and XY designed the study and wrote the protocol. JD managed the literature searches and analyses. JD and JX undertook the statistical analysis. LS wrote the first draft of the manuscript. LS and JD contributed equally. All authors contributed to the article and approved the submitted version.

## Funding

This work was supported by the National Natural Science Foundation of China (grant number 71974196).

## Conflict of Interest

The authors declare that the research was conducted in the absence of any commercial or financial relationships that could be construed as a potential conflict of interest.

## Publisher’s Note

All claims expressed in this article are solely those of the authors and do not necessarily represent those of their affiliated organizations, or those of the publisher, the editors and the reviewers. Any product that may be evaluated in this article, or claim that may be made by its manufacturer, is not guaranteed or endorsed by the publisher.
