# Supplementary material for: Rumination’s Role in Second Victim Nurses’ Recovery From Psychological Trauma: A Cross-Sectional Study in China
Source: Front Psychol. 2022 May 3;13:860902. doi: 10.3389/fpsyg.2022.860902 (PMC9110963; doi:10.3389/fpsyg.2022.860902)
Supplement: Supplementary file 1 [file Data_Sheet_1.pdf]

Supplementary Materials

Supplementary Table 1. Participant Demographics (*n*=233).

**Variable**	**Frequency (n)/percentage (%)**
**Gender**	
Male	8(3.43%)
Female	225(96.57%)
**Age**	
21-30	76(32.62%)
31-40	137(58.80%)
41-50	16(6.87%)
51-60	4(1.72%)
**Hospital level**	
Primary hospital	4(1.72%)
Secondary hospital	12(5.15%)
Tertiary hospital	217(93.13%)
**Affiliated department**	
Internal medicine	103(44.21%)
Surgery	68(29.18%)
Other	62(26.61%)
**Working years**	
1-5	41(17.60)
6-10	72(30.90%)
11-20	96(41.20%)
>20	24(10.30%)
**Education**	
College degree	30(12.88%)
Bachelor's	193(82.83%)
Master's or above	10(4.29%)
**Professional titles**	
Nurse	21(9.01%)
Nurse practitioner	116(49.79%)
Nurse-in-charge	87(37.34%)
Associate Professor in Nursing	9(3.86%)
**Marital status**	
Unmarried	49(21.03%)
Married	184(78.97%)
**Employment type**	
Regular employee	124(53.22%)
Contract staff	100(42.92%)
Other	9(3.86%)
**Monthly income (RMB yuan)**	
<2000	4(1.72%)
2000-4999	50(21.46%)
5000-7999	69(29.61%)
>8000	110(47.21%)

**Heard of the term "Second Victim"**	
Yes	62(26.61%)
No	171(73.39%)
**Adverse events experienced**	
Personally experienced	129(55.36%)
Only Witnessed	104(44.64%)

Supplementary Table 2. Descriptive Statistics of SV Nurses' Psychological Trauma, Rumination and PTG (*n*=233).

**Variable**	***Min***	***Max***	***M*±*SD***
**SV**			
Physical distress	2	5	4.16±.94
Psychological distress	2	5	4.65±.56
Practice-related distress	1	5	3.10±.99
Non-work related support	1	5	2.23±1.05
Colleague support	1	4	1.78±.71
Management support	1	5	1.96±.75
**Rumination**			
Active rumination	10	40	27.43±7.80
Invasive rumination	10	40	27.24±8.36
Total score	21	80	54.67±15.48
**PTG**			
Insights on life	6	30	22.80±4.60
Personal power	3	15	11.40±2.41
New possibilities	4	20	14.22±3.54
Relationship with others	3	15	10.80±2.57
Self-transformation	5	25	17.28±4.21
Total score	21	105	76.18±16.00

Supplementary Table 3. SV Nurses' Support Needs (*n*=233).

**Questionnaire items**	***M* ±*SD***	***Cumulative frequency (%)***
NO.4 The opportunity to schedule a time with a counsellor at my hospital to discuss the event	1.41±.566	96.1
NO. 8 A confidential way to get in touch with someone 24 hours a day to discuss how my experience may be affecting me	1.41±.573	95.7
NO.6 An employee assistance program that can provide free counselling to employees outside of work.	1.47±.609	94.8
NO. 3 A respected peer to discuss the details of what happened.	1.45±.601	94.4
NO. 7 A discussion with my manager or supervisor about the incident.	1.54±.649	92.3
NO. 5 A specified peaceful location that is available to recover and recompose after the occurrence of one of these types of events.	1.52±.644	91.8
NO. 2 Establishment of a fair culture or exemption mechanism in the hospital.	1.54±.688	90.6
NO. 1 The ability to immediately take time away from my unit for a little while.	2.18±1.070	65.2

Supplementary Table 4. Correlation Among Psychological Trauma, Rumination and PTG.

	**1**	**2**	**3**	**4**	**5**	**6**	**7**	**8**	**9**	**10**	**11**	**12**	**13**	**14**	**15**	**16**
**1** SV psychological trauma	1	.532**	.403**	.481**	.498**	.412**	.453**	.378**	.509**	.465**	-.135*	-.228**	-.207**	-.163*	0.002	-.154*
		0	0	0	0	0	0	0	0	0	0.04	0	0.001	0.012	0.972	0.019
**2** Physical distress		1	.641**	.441**	-0.125	-.287**	-.172**	.494**	.587**	.566**	.216**	0.049	.161*	.162*	.297**	.196**
			0	0	0.057	0	0.009	0	0	0	0.001	0.461	0.014	0.013	0	0.003
**3** psychological distress			1	.337**	-0.107	-.259**	-.179**	.337**	.403**	.388**	0.069	-0.06	0.026	0.078	.151*	0.06
				0	0.103	0	0.006	0	0	0	0.293	0.365	0.696	0.233	0.021	0.36
**4** Practice-related distress				1	-0.117	-0.062	-0.091	.373**	.518**	.468**	-0.101	-.207**	-0.108	-0.083	-0.01	-0.108
					0.076	0.342	0.168	0	0	0	0.123	0.001	0.099	0.205	0.879	0.101
**5** Non-work related support					1	.428**	.369**	-0.072	-0.046	-0.061	-.157*	-0.115	-.247**	-.187**	-.161*	-.186**
						0	0	0.275	0.485	0.353	0.016	0.08	0	0.004	0.014	0.004
**6** Colleague support						1	.518**	-0.122	-0.105	-0.118	-.189**	-.137*	-.254**	-.229**	-.195**	-.220**
							0	0.064	0.111	0.072	0.004	0.036	0	0	0.003	0.001
**7** Management support							1	-0.064	-0.022	-0.044	-.235**	-.187**	-.247**	-.219**	-.179**	-.231**
								0.33	0.743	0.504	0	0.004	0	0.001	0.006	0
**8** Active rumination								1	.835**	.955**	.166*	0.026	.134*	.132*	.276**	.163*
									0	0	0.011	0.692	0.041	0.044	0	0.013
**9** Invasive rumination									1	.961**	0.104	-0.048	0.034	0.063	.212**	0.087
										0	0.114	0.47	0.606	0.335	0.001	0.185
**10** Total of Rumination										1	.140*	-0.013	0.086	0.101	.254**	.129*
											0.033	0.849	0.191	0.125	0	0.049
**11** Insight on life											1	.876**	.846**	.817**	.795**	.950**
												0	0	0	0	0
**12** Personal power												1	.776**	.717**	.658**	.867**

													0	0	0	0
**13** New possibility													1	.823**	.792**	.925**
														0	0	0
**14** Relationships with others														1	.761**	.892**
															0	0
**15** Self-transformation															1	.883**
																0
**16** Total of PTG																1

Note. ***. Correlation is significant at the 0.01 level (2-tailed); *. Correlation is significant at the 0.05 level (2-tailed).*

Supplementary Table 5. The Predictive Effect of SV Nurses' Psychological Trauma and Rumination on PTG

Dependent variable	Predictor variable	*R*	R^2	*F*	*B*	*t*
Post-traumatic growth	Psychological trauma<br>Active Rumination<br>invasive Rumination	.285	.081	6.725***	-.251<br>. 259<br>.001	-3.398***<br>2.244*<br>.991

*Note. * and *** represent statistical significance at the levels of 0.05 and 0.001, respectively.*
